# Supplementary material for: Developing and evaluating a brief, socially primed video intervention to enable bystander cardiopulmonary resuscitation: A randomised control trial
Source: PLoS One. 2024 Jul 5;19(7):e0297598. doi: 10.1371/journal.pone.0297598 (PMC11226058; doi:10.1371/journal.pone.0297598)
Supplement: S1 Appendix — (DOCX) [file pone.0297598.s001.docx]

## S1 Appendix

### Summary of UBV script development and testing

### Study 1a: Creating the UBV Script

This study was completed in collaboration with Chiara Addison and Emma Brown, undergraduate students at the Department of Psychology in the University of Edinburgh.

#### Design:

A 1 x 2 cross-sectional research design was employed. Two short CPR familiarisation video scripts were developed, an experimental script that included language choices that evoked salience of Scottish identity, and a control script that had no social identity language.

#### Participants:

90 CPR-naive participants were recruited and randomly assigned to either the experimental (*n* = 43) or control (*n* = 47) group. 58.4% of the sample was female, 32.5% of the sample was male, and 2.7% of the sample identified as another gender. 56.5% of the sample was aged 18 - 24 years, 17.3% of the sample was aged 25 - 34 years, 12.2% of the sample was aged 35 - 54 years, and 7.1% of the sample was aged 55 - 85 years. Participants then provided online feedback on their assigned script. The exclusion criteria were being under 18 years, living outside Scotland, and not completing the survey.

#### Materials:

Participants completed a survey battery that examined basic demographic information, social identity, and attitudes to CPR.

#### Procedure:

Participants were recruited online via Facebook. Participation took place completely online. Participants completed the demographic information questionnaire before reviewing their assigned CPR video script. They then completed the remaining questionnaires. The process took approximately 10-minutes to complete.

#### Analyses:

Confirmatory factor analyses were conducted to determine the questionnaire items that underlay the variables of interest (shared social identity with Scottish people, shared social identity with the CPR instructor, efficacy, and expected support from Scottish people). Reliability analyses and manipulation checks were also completed. Bootstrapped mediation analyses were used to examine the relationships between the variables of interest. The findings were then implemented into the next iteration of the UBV.

### Results

Using confirmatory factor analysis and bootstrapped mediation analyses, Study 1a sought to ascertain whether the use of collectivist language would increase participants’ self-reported efficacy relevant to performing CPR via shared social identity with the CPR instructor. Additionally, this study examined whether raising the salience of Scottish identity would be associated with expected support from Scottish people in an emergency, and if this relationship would be mediated by a sense of shared social identity between Scottish people.

S1 Figure 1 demonstrates the relationship between the group condition (social identity language vs no social identity language) and efficacy, as mediated by shared social identity with the CPR instructor. The relationship between group condition and efficacy was not found to be mediated by shared social identity with the CPR instructor (indirect effect: β = .08, CI [-.001, .24]). However, this indirect effect was positive and approached significance. A positive significant pathway effect was observed between group condition and shared social identity with the instructor (β = .27, CI [.07, .47]). A positive but non-significant pathway effect was found between shared social identity with the instructor and efficacy (β = .30, CI [-.04, .66]).

*
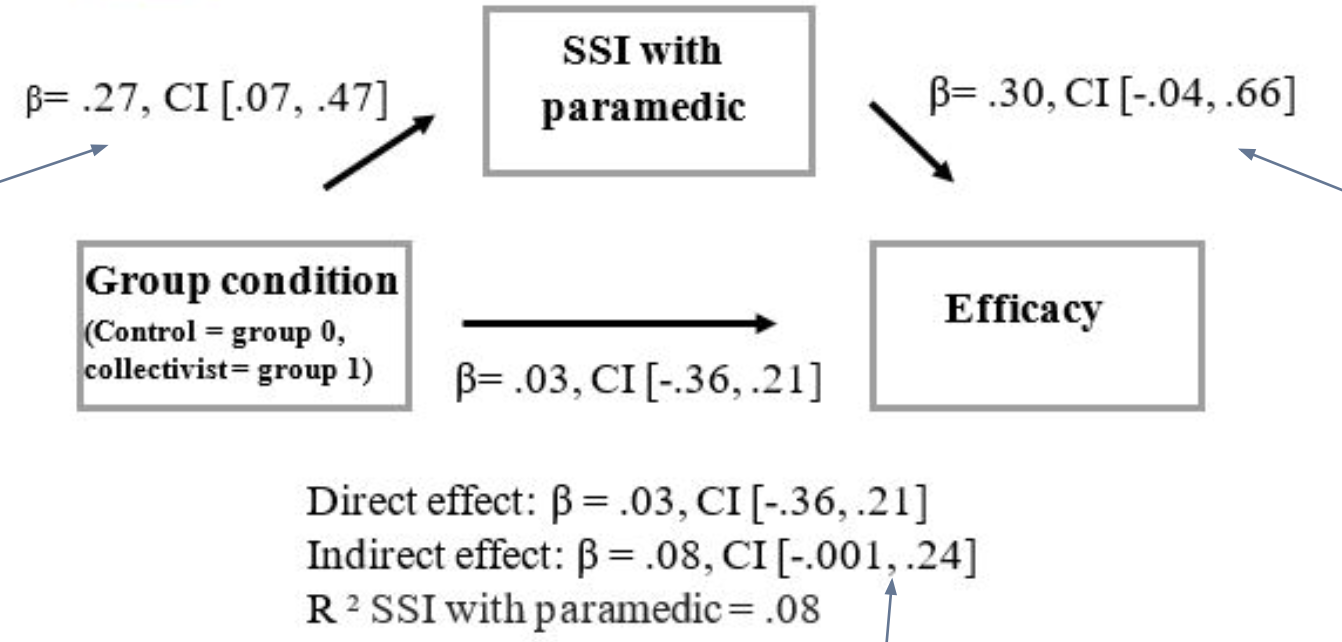
*

**instructor**

**instructor**

*S1 Fig 1. Mediation analysis of the relationship between group condition, efficacy, and shared social identity with the video instructor*.

S1 Figure 2 demonstrates the relationship between group condition and expected support from Scottish people in an emergency, as mediated by shared social identity with Scottish people. The relationship between group condition and expected social support was not found to be mediated by shared social identity with Scottish people (β = 0.11, *p* = 0.09 [-0.007, 0.240]. However, this indirect effect was found to be positive, despite not reaching significance. A positive but non-significant pathway effect was observed between group condition and shared identity with other Scottish people (β = 0.28, *p* = 0.07). A significant positive pathway effect was observed between shared social identity with Scottish people and expected support from Scottish people (β = 0.38, *p* = 0.002).

Positive, significant effect


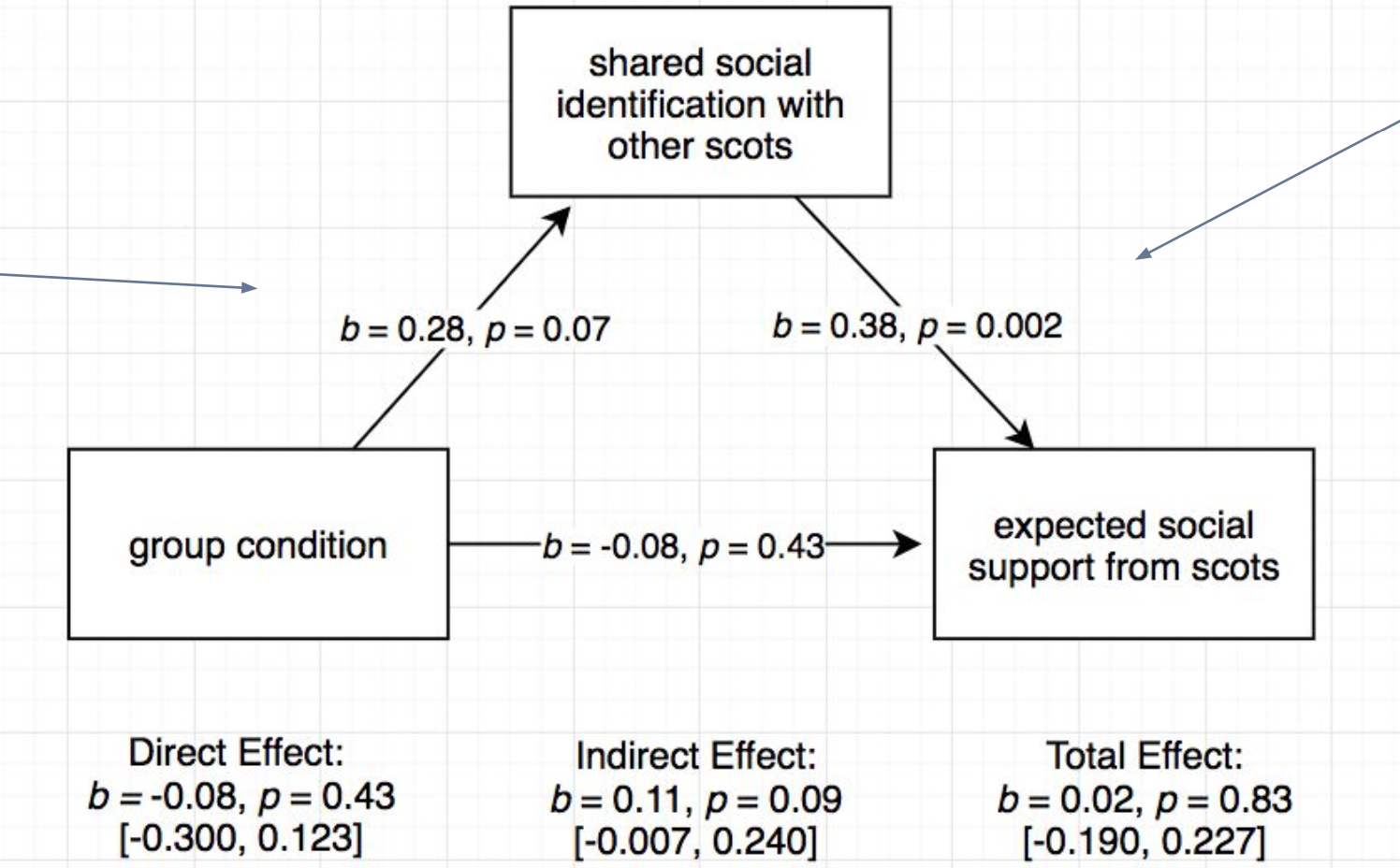


Positive, non- significant

effect

*S1 Fig 2. Mediation analysis of the relationship between group conditions, expected social support from other Scottish people, and shared social identity with other Scottish people.*

### Study 1b: Refining the UBV script, creation of the video, testing of CPR knowledge

This study was completed in collaboration with Jennifer Dang Guay, postgraduate student at the Department of Psychology in the University of Edinburgh.

#### Design:

A 2 x 2 between subjects experimental design was employed. The scripts from Study 1a were further refined using participant feedback and then made into four instructional videos. These were i) long form video, no social priming language (LA); ii) short form video, no priming language (SA); iii) long form video, with priming language (LP); iv) short form video, with priming language (SP).

#### Participants:

223 participants were recruited online via Prolific. They were then assigned to one of the four video conditions: LA (*n* = 57), SA (*n* = 56), LP (*n* = 56), SP (*n* = 54). 74% of the sample was female, 24% was male, and 1.5% identified as other genders. 59% of the sample was aged 18 - 35 years, 34% of the sample was aged 35 - 54 years, and 7.3% of the sample was aged 55 - 85 years. Exclusion criteria included being under 18 years, living outside of Scotland, and failing the manipulation and attention checks.

#### Materials:

Participants completed a survey battery (see Appendix B for further details) that examined demographic characteristics, prior CPR knowledge, social identity, and attitudes towards CPR.

#### Procedure:

Data were gathered online via Prolific. Participants were randomly assigned to one of the four video conditions. Once participants watched their assigned video, they completed the survey materials.

#### Analysis:

Wilcoxon-Mann-Whitney tests for independent samples were employed to examine average differences between the different conditions. 2-way independent ANOVA using non-parametric bootstrapping was used to examine the relationship between each video condition and willingness to perform CPR, perceived clarity of the videos, and perceived provision of sufficient practical information. Findings were then implemented into the next iteration of the UBV.

### Results

Wilcoxon-Mann-Whitney tests for independent samples and 2-way independent ANOVA were used to examine differences between the four video conditions (SP = short length, priming language; SA = short length, no priming language; LP = long length, priming language, LA = long length, no priming language). Significantly higher Scottish social identity scores were found in the SP group in comparison to the SA group (*W* = 1969, *p* < .01). Significantly higher Scottish social identity scores were also found in the LA group as opposed to the SA group (*W* = 2191, *p* < .001).

With regard to willingness to perform CPR, 2-way ANOVA revealed that ratings were similar across all groups (*p*  > .05), suggesting that video length did not impact a participant’s willingness to perform CPR. 2-way ANOVA using non-parametric bootstrapping with 2000 resamples demonstrated that there were significant differences between the long and short video groups for the variables of perceived clarity and provision of sufficient information, but that these differences only just reached significance thresholds. Perceived clarity scores were found to be significantly higher in the long video groups (*B* = 0.17, *SE* = 0.07, CI [0.02, 0.31]). This same trend was observed in terms of perceived provision of sufficient practical information from the videos, in which the long length videos had significantly higher scores (*B* = 0.25, *SE* = 0.09, CI [0.07, 0.43]).

## 
