# Supplementary material for: Developing and evaluating a brief, socially primed video intervention to enable bystander cardiopulmonary resuscitation: A randomised control trial
Source: PLoS One. 2024 Jul 5;19(7):e0297598. doi: 10.1371/journal.pone.0297598 (PMC11226058; doi:10.1371/journal.pone.0297598)
Supplement: S2 Appendix — (DOCX) [file pone.0297598.s002.docx]

## S2 Appendix

### Laerdal QCPR Metrics

CPR quality was reported as measured by the Laerdal QCPR App. This app measures CPR quality across the areas of compression depth and rate in order to form both an overall compression score and a total composite score of CPR performance. The CPR metrics that inform these scores are compression depth/release measured in millimetres, compression speed, time on the chest doing active compressions, total number of compressions, and average number of compressions per minute. The QCPR app can also measure metrics relevant to ventilations, however this functionality was not used in the current study. The overall compression score and an overall CPR quality score is calculated using a non-binary scoring algorithm developed by Laerdal Medical. To learn more about this scoring algorithm, see the links to Laerdal Medical’s website below.

<https://laerdal.com/us/support/scoring/>

<https://laerdal.force.com/HelpCenter/s/article/QCPR-App-feedback-symbols-and-scoring>
