# Supplementary material for: Developing and evaluating a brief, socially primed video intervention to enable bystander cardiopulmonary resuscitation: A randomised control trial
Source: PLoS One. 2024 Jul 5;19(7):e0297598. doi: 10.1371/journal.pone.0297598 (PMC11226058; doi:10.1371/journal.pone.0297598)
Supplement: Attachment — Submitted filename: Response_To_Reviewers20240506.docx [file pone.0297598.s003.docx]

Submission PONE-D-24-00549

Response to Reviews

**6^th^ May 2024**

Dear Dr Wampler,

Thank you for giving us the opportunity to resubmit a revised copy of the manuscript “Developing and evaluating a brief, socially primed video intervention to enable bystander cardiopulmonary resuscitation: A randomised control trial” for *PLOS ONE*. We appreciate the valuable feedback you and the reviewer provided on the paper. We have incorporated these improvements into the current draft of the manuscript, which are highlighted with Track Changes, Please see below our responses to each feedback point in blue font. Please note that line numbers correspond to the Track Changes manuscript draft:

**Reviews’ comments to the authors:**

1. Manuscript page 10, line 57: hyphenate information-heavy?

**Author response:** Thank you for pointing this out. “Information-heavy” has been hyphenated for clarity and grammatical correctness (Line 88).

2. Manuscript page 12, line 105-110: Rationale for different training equipment (manikin v bag) understandable, but ideally as few variables as possible would have been changed – granted this is effectively an intention-to-treat paradigm, wherein the entire training model is being compared to another, rather than only the video component. Hoping this is discussed later. (edit: I don’t believe it was mentioned)

**Author response:** We agree with the reviewer that the rationale for using a bag manikin in the ultra-brief condition could be more explicitly explained in the text. As the reviewer points out, the intervention being tested was the training model in its entirety (i.e. both the manikin and the video as a unit), rather than just the familiarisation videos in isolation. As accessibility to CPR familiarisation is a core tenet of our ultra-brief intervention, and compression practice using a manikin is a key quality indicator of resulting CPR skills, we decided to use the freely availably/printable bag manikin for our ultra-brief condition. This bag manikin fit more with the ethos of this intervention, as inflatable Mini Anne manikins usually come with a cost. We have added a passage to the background section to highlight this rationale (Lines 134, 142-144; 167-168).

3. Results section of the abstract: please rework this just a bit, the current version lacks the detail presented in the main body of the manuscript.

**Author response:** Thank you for this feedback, we have reworked the abstract to provide a more detailed summary of the results section that is more in line with the results presented in the main text. (Lines 21-50)

4. Results of the main manuscript: authors state that eight subjects were excluded due to technical issues. Please report from how many were excluded from each study arm.

**Author response:** We agree that the data lost to technical issues could be more clearly stated. We have specified the number of participants excluded from the QCRP dataset for each condition due to technical issues with our QCPR manikin. Prior to submitting the manuscript for publication, data from one participant that had been lost to a technical issue was recovered. This passage was not updated to reflect this, and so has now been updated with the correct details on numbers of participants excluded per study arm (Lines 228-230).

5. Table 1: Worth noting in the table that the ages, though visibly skewed older in the LFV group, does not meet statistically significant difference (at least according to my napkin-math Fisher’s Exact), nor does the employment/student category. The first impulse I had upon looking at it was that the younger cohort got the short-attention-span friendly video – but speaking to nonsignificant difference may allay some of that objection.

**Author response:** The reviewer raises a good point here – we conducted Fisher’s Exact Tests on all demographic variables to ensure there were no significant inter-group differences between our conditions. We have highlighted this more clearly in Table 1. (Lines 225-226)

6. Manuscript page 15, line 298: Something of a quibble, but when using nonparametric tests for everything else, working in Cohen’s d (a parametric test calculated using means and SDs) seems incongruous. While only the exceptionally nerdy (e.g., me) would notice or care – res ipsa loquitur for most of the findings – I’d be remiss not to mention it. Rank biserial is the nonpar equivalent, if wishing to modify. Or dismiss out of hand because the end state is almost certainly the same.

**Author response:** Thank you for this informative feedback – the reviewer is correct that a non-parametric effect size would make more sense in the context of our analysis. While the reviewer is correct that this did not change the end state, we want to be consistent with our analysis, and so have replaced our Cohen’s d values with rank-biserial values (Lines 335-336; 344-345; 348; 366-367).

7. Tables 2,3: There are a lot of tests being run here, and no mention of multiple comparisons adjustments against minimum three hypotheses, and at least 19 tests identifiable amongst the tables. Granted, the consequences of type I error in this scenario are profoundly low – just something to consider regarding internal consistency.

**Author response:**  The reviewer raises an important point here regarding multiple comparisons and Type I Error. When planning our analysis, this was something we considered carefully. As we were working with 2 separate datasets (the QCPR participants and the post-intervention survey datasets), we felt that our analyses were adequately robust against Type I Error without additional corrections for multiple comparisons. We also considered that controlling for multiple comparisons in this case could result in an overly conservative thresholds for significance that may end up masking meaningful findings. However, we recognise that the risk of Type I Error in this case is not zero, and therefore have added Benjamini-Hochberg-corrected *p-*values to the Results section to be fully rigorous in our analyses. This method was chosen as it is suitable for controlling the False Discovery Rate when conducting multiple comparisons with non-parametric tests, while preserving statistical power (Lines 332-333; 344-345; 348; 356; 360-364; 366-367; 372; 373; 384-386; 429-431; 470-471).

8. Figure two is too low in resolution to be publication quality, please provide a better version.

**Author response:** We apologise for this low resolution figure; we have attached a higher resolution 300 DPI version of our Figure 2 that we hope will suffice for publication quality.
