# Supplementary material for: Developing and evaluating a brief, socially primed video intervention to enable bystander cardiopulmonary resuscitation: A randomised control trial
Source: PLoS One. 2024 Jul 5;19(7):e0297598. doi: 10.1371/journal.pone.0297598 (PMC11226058; doi:10.1371/journal.pone.0297598)
Supplement: Attachment — Submitted filename: Response_To_Reviewers20240607.docx [file pone.0297598.s004.docx]

Submission PONE-D-24-00549R1

Response to Reviews

**7^th^ June 2024**

Dear Dr Wampler,

Thank you for giving us the opportunity to resubmit a revised copy of the manuscript “Developing and evaluating a brief, socially primed video intervention to enable bystander cardiopulmonary resuscitation: A randomised control trial” for *PLOS ONE*. We appreciate the valuable feedback you and the reviewer provided on the paper. We have incorporated these improvements into the current draft of the manuscript, which are highlighted with Track Changes, Please see below our responses to each feedback point in blue font. Please note that line numbers correspond to the Track Changes manuscript draft:

**Reviews’ comments to the authors:**

1. Manuscript page 11, line 216: Embarrassed at not noticing this the first time around – the participants are not random people, but University students and staff. While the limitations section notes the COVID-related challenge, it does not currently examine problems with extrapolating the findings to the lay public at large. It serves as a fine proof of concept, and needs little more than a head-nod to the issue, but at the moment one can only say that amongst those already selected for intelligence and reliability, UBV appears to serve the requirement.

**Author response:** Thank you for pointing this out. We have added a passage to the limitations section that more explicitly outlines that the study employed a university sample and that therefore results cannot be generalised to the wider public. We tied this into our recommendation that future research explore the utility of UBV CPR interventions with a more representative sample for the purposes of generating more generalisable findings (lines 425-430).

2. Manuscript page 21, line 392, and line 395: In the spirit of ongoing pedantry on this reviewer’s part, the authors are asked to consider that “i.e.” (id est – that is to say – a more specific paraphrase or revealed implication) be replaced with “e.g.” (exempli gratia – free example/for the sake of example, ideally out of a greater unstated list) in these cases. I.e. is used correctly (in this reviewer’s estimation) higher up in manuscript page 5, line 88, while in manuscript page 5, line 96, the problem is avoided by spelling out “such as.” I realize I could have said nothing and the outcome would be unchanged; observations like this are not a hit at parties. Overall, the language of the manuscript is a pleasure to read.

**Author response:** On the contrary, we greatly appreciate the reviewer’s attention to detail! “i.e.” has been changed to “e.g.” in both cases for grammatical correctness (lines 392; 395).

3. General comment: If I worked for the Scottish Ambulance Service, I would absolutely tell people I worked for the SAS and refuse to elaborate, claiming it was classified.

**Author response:**  The corresponding author now regrets not taking up a contract with the ambulance service.
